# A Simple Drug Delivery System for Platelet-Derived Bioactive Molecules, to Improve Melanocyte Stimulation in Vitiligo Treatment

**DOI:** 10.3390/nano10091801

**Published:** 2020-09-10

**Authors:** Karolina Vocetkova, Vera Sovkova, Matej Buzgo, Vera Lukasova, Radek Divin, Michala Rampichova, Pavel Blazek, Tomas Zikmund, Jozef Kaiser, Zdenek Karpisek, Evzen Amler, Eva Filova

**Affiliations:** 1 Institute of Experimental Medicine of the Czech Academy of Sciences, Videnska 1083, 142 20 Prague, Czech Republic; vera.sovkova@iem.cas.cz (V.S.); buzgo@inocure.cz (M.B.); vera.lukasova@iem.cas.cz (V.L.); radek.divin@iem.cas.cz (R.D.); michala.rampichova@iem.cas.cz (M.R.); eva.filova@iem.cas.cz (E.F.); 2 Department of Biophysics, 2nd Faculty of Medicine, Charles University, V Uvalu 84, 150 06 Prague, Czech Republic; evzen.amler@lfmotol.cuni.cz; 3 University Centre for Energy Efficient Buildings, Czech Technical University in Prague, Trinecka 1024, 273 43 Bustehrad, Czech Republic; 4 Central European Institute of Technology, Brno University of Technology, Purkynova 656/123, 616 00 Brno, Czech Republic; pavel.blazek@vutbr.cz (P.B.); tomas.zikmund@ceitec.vutbr.cz (T.Z.); jozef.kaiser@ceitec.vutbr.cz (J.K.); 5 Institute of Mathematics, Faculty of Mechanical Engineering, Brno University of Technology, Technicka 2, 616 69 Brno, Czech Republic; karpisek@fme.vutbr.cz

**Keywords:** vitiligo, melanocyte, platelets, electrospinning, centrifugal spinning

## Abstract

Vitiligo is the most common depigmentation disorder of the skin. Currently, its therapy focuses on the halting of the immune response and stimulation of the regenerative processes, leading to the restoration of normal melanocyte function. Platelet-rich plasma (PRP) represents a safe and cheap regenerative therapy option, as it delivers a wide spectrum of native growth factors, cytokines and other bioactive molecules. The aim of this study was to develop a simple delivery system to prolong the effects of the bioactive molecules released from platelets. The surface of electrospun and centrifugally spun poly-ε-caprolactone (PCL) fibrous scaffolds was functionalized with various concentrations of platelets; the influence of the morphology of the scaffolds and the concentration of the released platelet-derived bioactive molecules on melanocytes, was then assessed. An almost two-fold increase in the amount of the released bioactive molecules was detected on the centrifugally spun vs. electrospun scaffolds, and a sustained 14-day release of the bioactive molecules was demonstrated. A strong concentration-dependent response of melanocyte to the bioactive molecules was observed; higher concentrations of bioactive molecules resulted in improved metabolic activity and proliferation of melanocytes. This simple system improves melanocyte viability, offers on-site preparation and is suitable for prolonged topical PRP administration.

## 1. Introduction

Vitiligo is the most common depigmentation disorder affecting approximately 1% of the population in the US and Europe; however, the occurrence ranges from 0.1% to 2% worldwide [1,2]. The disease is characterized by an acquired, idiopathic and progressive loss of pigment in hair and/or skin, secondary to the loss of functional melanocytes [3]. Although vitiligo is not a life-threatening condition, it can result in depression, anxiety, poor body image and social difficulties, affecting the quality of life of patients [4,5]. The currently available treatment options focus on the re-pigmentation of the existing lesions and include pharmacotherapy (such as topical corticosteroids) [6], narrow-band UVB (NB-UVB) phototherapy [7] or use of an excimer laser [8]. Furthermore, in recalcitrant lesions, autologous melanocyte transplantation may be used. Taking into account the not yet fully understood, diverse and complex etiopathogenesis of the disease, the outcomes are unsatisfactory [6].

Recently, platelet-rich plasma (PRP) has been successfully used in vitiligo therapy, resulting in improved and sustained repigmentation [9,10]. Platelets, owing to the high content of native growth factors, chemokines and cytokines in their α-granules, represent a cost-effective and possibly autologous reservoir of bioactive molecules, such as vascular endothelial growth factor (VEGF), fibroblast growth factor (FGF), platelet-derived growth factor (PDGF), transforming growth factor beta (TGF-β) or Regulated upon Activation, Normal T Cell Expressed and Secreted (RANTES) [11,12,13]. Platelet products have recently been used in various applications in dermatology, including face rejuvenation procedures [14,15], hair regrowth [16] or scar management [17,18].

The half-life of the growth factors is generally very short, limiting their bioavailability [19,20]. Therefore, drug delivery systems combining a biomaterial matrix protecting the soluble growth factors and preventing their fast clearance and/or diffusion from the site of administration, would control their spatial–temporal release [21]. Nanofibers could be suitable for use as a biomaterial. Their unique properties, such as high volume-to-surface ratio, porous structure, and nanoscale microarchitecture, predetermine them for tissue engineering applications [22]. The most common large-scale techniques of nanofiber fabrication include electrospinning (using electrostatic forces to produce fibers) and centrifugal spinning (producing fibers using centrifugal forces) [23,24,25]. As a postproduction modification, surface adhesion is the simplest method for scaffold functionalization; however, the loading efficiency and release rate may be quite variable and difficult to optimize. Furthermore, the adsorbed molecules may be released quickly, and such systems are better suited for short-term delivery. Several types of bioactive molecules have been successfully adsorbed to the surface of nanofibers, such as growth factors for the acceleration of tissue regeneration in hernia repair [26], antibiotics for the prevention of post-op adhesions [27] or liposomes loaded with growth factors to promote cell proliferation [28,29].

In this study, a simple drug delivery system consisting of native platelets immobilized to the surface of the fibrous scaffolds was tested. The nanofibers were fabricated from poly-ε-caprolactone (PCL) using two different techniques, electrostatic and centrifugal spinning. Various concentrations of platelets, the differing morphology of the fibrous layers, and the effect of the released molecules, were tested using melanocytes in vitro, as the role of platelet-rich plasma in melanocyte stimulation is not fully understood.

## 2. Materials and Methods

### 2.1. The Fabrication of Electrospun and Centrifugally Spun Scaffolds

The electrospun PCL scaffolds were fabricated using the Nanospider™ NS 500 device (Elmarco, Liberec, Czech Republic). To perform the electrospinning process, a 24% (*w*/*v*) solution of PCL (MW 45,000; Sigma Aldrich, St. Louis, MO, USA), dissolved in a mixture of chloroform and ethanol (volume ratio 9:1) was used. The polymer solution was connected to the high-voltage source (Spellman HV, Hauppauge, NY, USA). The voltage used for electrospinning was 70 kV, the distance was set at 35 cm and the polymer flow rate was 50 mL/h. The solution was electrospun using a needleless wire electrode and the nanofibers were deposited on a non-woven, supporting Spunbond textile (Pegas Nonwovens, Znojmo, Czech Republic). The conditions during the process were maintained at 23° ± 2 °C and 60 ± 15% relative humidity.

The centrifugally spun PCL scaffolds were fabricated using a centrifugal spinning device (Cyclone L-1000D Forcespinning^®^ device; FibeRio, McAllen, TX, USA). To perform the centrifugal spinning process, a 40% (*w*/*v*) solution of PCL (MW 45,000; Sigma Aldrich, St. Louis, MO, USA) dissolved in a mixture of chloroform and ethanol (volume ratio 9:1) was used. An orifice G30 at a rotation speed of 6000× *g*, was used to prepare the layers. The formed fibers were deposited on a non-woven textile (Pegas Nonwovens, Czech Republic) using a vacuum-assisted deposition.

### 2.2. Characterization of the Scaffolds Using Sub-µCT

The samples were scanned using the Rigaku Nano3DX device (Rigaku Corporation, Tokio, Japan) with a Cu target operating at 30 kV, 3300 × 2500 px2 X-ray CCD camera and 0.27 × 0.27 µm^2^ pixel size. The exposure time was set at 12 s to reach a sufficient amount of signal. 800 projections were acquired over 180° rotation. Binning 2 was set (information from 2 × 2 px^2^ area was summed) to decrease data noise and measurement time. The voxel size of (0.54 µm) 3 was reached. The phase retrieval algorithm [30,31] was used to enhance the contrast between the fibers and background. Data were reconstructed using Rigaku reconstruction software. The analysis was performed in software VGStudio MAX 3.2 (Volume Graphics GmbH, Heidelberg, Germany). The advanced method of thresholding based on local gray valued differences, was applied to segment the fibers. The volume of the cells between fibers was analyzed using a Foam structure analysis module, with a merge threshold set to 5% and fast precision [32].

### 2.3. The Functionalization of Fibrous Scaffolds with Native Platelets

The surface of the prepared electrospun and centrifugally spun PCL samples was functionalized with different concentrations of platelets. The fabricated PCL samples (diameter 6 mm) were punched out of the fibrous mats. The samples were sterilized using 70% ethanol and rinsed with a phosphate buffered saline (PBS) three times. Fresh human leukocyte-poor platelet concentrate derived from a buffy-coat (in additive solution) was obtained from the Blood Transfusion Service (Šumperk, Czech Republic). The bag was prepared from the blood of four donors (All subjects gave their informed consent for inclusion before they participated in the study. The study was conducted in accordance with the Institute of Experimental Medicine CAS, and the protocol was approved by the Ethics Committee of 2020/04). The platelet concentration in the bag was 967 × 10^9^ platelets/L. The tested platelet concentrations are given in Table 1. The solutions containing the appropriate concentration of platelets were prepared by diluting the platelet concentrate in SSP+™ additive solution (Macopharma, Tourcoing, France). In the event of a solution with a platelet concentration being higher than the platelet concentration in the bag, the platelet concentrate was firstly centrifuged (3100× *g* for 10 min) and then re-suspended in the appropriate volume of SSP+™ to obtain the desired platelet concentration. Finally, 50 μL of the prepared solutions were added to the sterile nanofibrous samples in a 96-well plate. The samples were incubated for 2 h at a temperature of 22 °C. The samples were subsequently washed with PBS and placed into new wells. Plain PCL scaffolds served as controls (E0 for electrospun PCL samples, C0 for centrifugally spun PCL samples).

### 2.4. Characterization Using Scanning Electron Microscopy

The structure of the fabricated scaffolds was visualized using scanning electron microscopy (SEM). The dry samples were sputter coated with gold using a Q150RS device (Quorum Technologies, Lewes, UK). A Vega3 SBU (Tescan, Brno, Czech Republic) microscope was used for image acquisition. The fiber diameter and pore size of the prepared samples was determined using ImageJ software (version 1.53a, National Institutes of Health, Bethesda, MD, USA) from at least 200 independent measurements.

To visualize the platelets adhered to the surface of the scaffolds, the samples were fixed using 2.5% glutaraldehyde (3 h, 4 °C; Sigma-Aldrich, St. Louis, MO, USA) following the 2-h incubation of the fibrous samples with the respective platelet solutions. Subsequently, the samples were dehydrated with ethanol changes (35%, 48%, 70%, 96% and 100% ethanol). Hexamethyldisilazane (Sigma-Aldrich, MO, USA) was used to remove any residual water from the samples. The samples were sputter-coated with gold (app. 30 nm) and visualized using Vega 3 SBU microscope (Tescan, Czech Republic).

### 2.5. Analysis of Bioactive Molecules Contained in Platelets

To characterize the bioactive molecules contained in platelets, platelet lysate was firstly prepared. The samples underwent three freeze/thaw cycles (−80 °C/37 °C) and were centrifuged to discard the cellular debris. The Bio-Plex 200 Multiplex System (Bio-Rad Laboratories, Hercules, CA, USA) was then employed. The commercially available cytokine panel (Bio-Plex Pro™ Human Cytokine 27-plex Assay, Bio-Rad Laboratories, Hercules, CA, USA) was used, in accordance with the manufacturer’s instructions. The assay allows multiple bioactive molecules to be quantified simultaneously in one well. The following analytes were quantified: interleukin-1b (IL-1b), interleukin 1ra (IL-1ra), interleukin-2 (IL-2), interleukin-4 (IL-4), interleukin-5 (IL-5), interleukin-6 (IL-6), interleukin-7 (IL-7), interleukin-8 (IL-8), interleukin-9 (IL-9), interleukin-10 (IL-10), interleukin-12 (IL-12), interleukin-13 (IL-13), interleukin-15 (IL-15), interleukin-17 (IL-17), granulocyte-colony stimulating factor (G-CSF), granulocyte-macrophage colony stimulating factor (GM-CSF), interferon-gamma (INF-γ), tumor necrosis factor-α (TNF-α), monocyte chemoattractant protein-1 (MCP-1), CXCL10 chemokine (C-X-C motif ligand-10), macrophage inflammatory protein-1a and 1-b (MIP-1a, MIP-1b), RANTES, eotaxin, PDGF, basic fibroblast growth factor (bFGF), and VEGF. Furthermore, the enzyme-linked immunosorbent assay (ELISA) was used to determine the concentration of thrombospondin-1 (TSP-1) and TGF-β1 in the platelet lysate according to the manufacturer’s instructions (DuoSet^®^; R&D Systems, Minneapolis, MN, USA).

### 2.6. The Detection of Released Growth Factors

To determine the release profiles of growth factors from the platelet-functionalized samples, TSP-1 and TGF-β1 were chosen as model molecules. The samples were weighed (approximately 80 mg) and to each sample, 500 µL of PBS was added. The samples were kept at 37 °C. On days 1, 3, 7 and 14 of the experiment, the PBS was collected, frozen (−80 °C) and replaced by new PBS. The concentration of TSP-1 and TGF-β1 released from platelets adhered to the electrospun and centrifugally spun samples, was quantified by ELISA in accordance with the manufacturer’s instructions (DuoSet^®^; R&D Systems, Minneapolis, MN, USA).

### 2.7. Cell Culture and Seeding

Murine melan-a cell line (melanocytes) were purchased from the Welcome Trust Functional Genomics Cell Bank at St. George’s University of London. The cells were cultured in a humidified incubator (37 °C, 10% CO_2_ and 80–90% relative humidity) in RPMI-1640 medium supplemented with 10% fetal bovine serum (FBS; Sigma Aldrich, Cat. No. F7524, MO, USA), penicillin/streptomycin (100 IU/mL, 100 μg/mL respectively), 2 mM L-glutamine and 200 nM 12-O-tetradecanoylphorbol 13-acetate TPA (Sigma-Aldrich, St. Louis, MO, USA) mixture. Sub-confluent cells were washed with PBS-EDTA (ethylenediaminetetraacetic acid) solution and treated with 0.05% trypsin/EDTA solution. The scaffolds were seeded with cells at a density of 17,850 cells/cm^2^. Half of the volume of the medium was changed every 7 days.

### 2.8. Cell Metabolic Activity Testing

On days 1, 3, 7, 10, and 14, the metabolic activity of melanocytes was determined using the MTS (3-(4,5-dimethylthiazol-2-yl)-5-(3-carboxymethoxyphenyl)-2-(4-sulfophenyl)-2H-tetrazolium) assay (CellTiter96^®^ AQueous One Solution Cell Proliferation Assay; Promega, Madison, WI, USA). To each scaffold, 20 μL of MTS solution and 100 μL of fresh medium were added. After incubation (3 h at 37 °C, 10% CO_2_, 80–90% relative humidity), the absorbance of the media was detected at 490 nm with a microplate reader (Infinite^®^ M200 PRO; Tecan, Männedorf, Switzerland). The background absorbance (690 nm) was subtracted from the measured data, along with the absorbance of a plain medium. Platelets contain mitochondrial enzymes and thus metabolize the MTS substrate. To exclude misrepresentation of the cell metabolic activity due to the presence of platelets on the surface of nanofibers, an MTS assay of samples with platelets was performed; the detected absorbance was subtracted from the measured data.

### 2.9. Cell Proliferation Testing

On days 1, 3, 7, and 14, melanocyte proliferation was determined using a fluorescence-based kit (Quant-iT™ PicoGreen^®^ dsDNA Assay Kit, Invitrogen Life Technologies, Carlsbad, CA, USA). The samples used for the MTS assay were transferred to a cell lysis buffer (10 mM Tris, 1 mM EDTA, 0.2% *v*/*v* Triton X-100). To facilitate the cell lysis and DNA release, the samples underwent three freeze/thaw cycles. In between the cycles, the samples were vortexed. Fluorescence intensity was detected using a microplate reader (Infinite^®^ M200 PRO; Tecan, Männedorf, Switzerland; λex = 485 nm, λem = 535 nm) and the DNA content was determined according to the λDNA calibration curve of the kit.

### 2.10. Cell Visualization Using Confocal Microscopy

Confocal microscopy was used to visualize the cells. The cells seeded on scaffolds were fixed with methanol (−20 °C), rinsed with PBS and stained. 3,3’-Dihexyloxacarbocyanine iodide (DiOC6(3)) was used to visualize the cellular membranes (Life Technologies, Carlsbad, CA, USA; 1 μg/mL in PBS, 30 min) and propidium iodide (Sigma-Aldrich, MO, USA; 5 μL/mL, 10 min) to visualize the cell nuclei. Between the incubations, the samples were rinsed with PBS. A Zeiss LSM 510 DUO confocal microscope was used for imaging (λex maximum = 488 nm, λem maximum = 501 nm for DiOC6(3); λex maximum = 536 nm, λem maximum = 617 nm for propidium iodide).

### 2.11. Statistical Analysis

The acquired data were statistically evaluated using SigmaStat 3.5 software (Systat Software Inc., San Jose, CA, USA). The statistical significance between a pair of groups was determined using ANOVA testing. Tukey’s comparative test was used for post-hoc analysis. The data are presented as a mean value plus/minus standard deviation. A value of *p* < 0.05 was considered statistically significant.

## 3. Results

### 3.1. Morphology Characterization of the Scaffold

**Figure 1:**
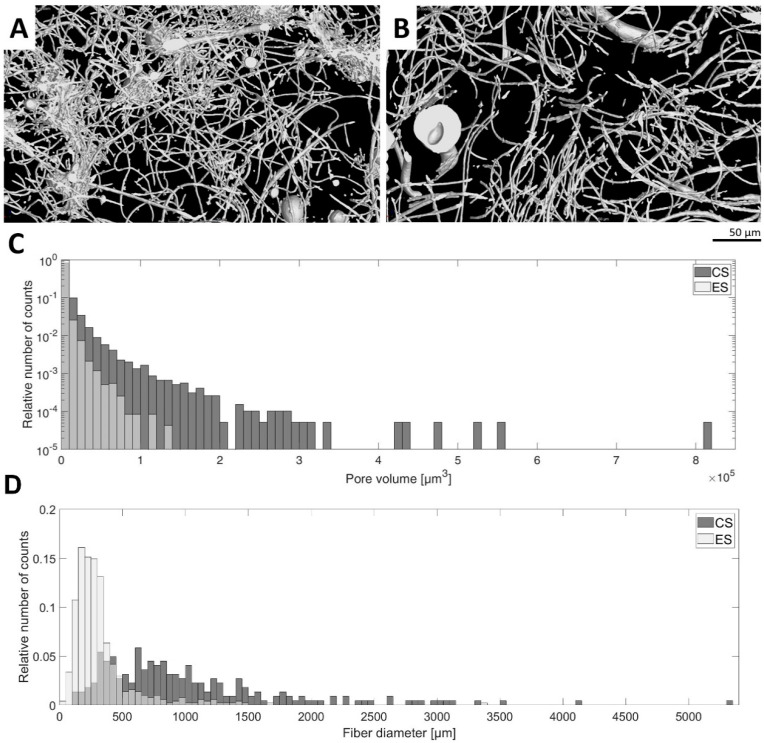
Morphology characteristics of the fibrous samples. The morphology of the electrospun (ES) and centrifugally spun (CS) samples was assessed using the sub-micron tomography (µCT) technique. (**A**)—3D rendering visualization of a 30 µm thick layer of the electrospun fibrous sample. (**B**)—3D rendering visualization of a 30 µm thick layer of the centrifugally spun fibrous sample. (**C**)—Histogram comparison of electrospun and centrifugally spun fibrous samples showing distributions of pore volume. (**D**)—Histogram comparison of electrospun and centrifugally spun samples showing the distributions of fiber diameter.

Nano-/microfibrous scaffolds were prepared using the two fiber-forming techniques: electrospinning and centrifugal spinning. Firstly, the morphology of the plain fibrous scaffolds without any platelets was studied. In the electrospun samples, we observed a bimodal distribution of nanofibers with a dominant thin nanofiber population (195 ± 85 nm) and a thick nanofiber population (875 ± 253 nm). In the case of centrifugally spun fibers, the thin nanofiber population (357 ± 153 nm) was much less abundant. The major population was microfibrous (1–2 µm) forming a bimodal nano-/microfibrous morphology. A histogram of fiber diameter distribution is given in Figure 1D. The X-ray computed sub-micron tomography (sub-µCT) evaluation of the fibrous samples showed that the morphology of the electrospun mesh was more compact than the centrifugally spun mesh based on the fiber density visualized in the 3D rendering (Figure 1A). This confirms the comparison of pore size median values, which are 458 µm^3^ for electrospun mesh and 2475 µm^3^ for centrifugally spun mesh. Histograms of pore volume frequency were compared using the Mann–Whitney test. A null hypothesis asserting that the medians of the two samples are identical was rejected against an alternative hypothesis that the median of the centrifugally spun sample is greater than that of the electrospun spun sample at *p*-value 0.0001.

**Figure 2:**
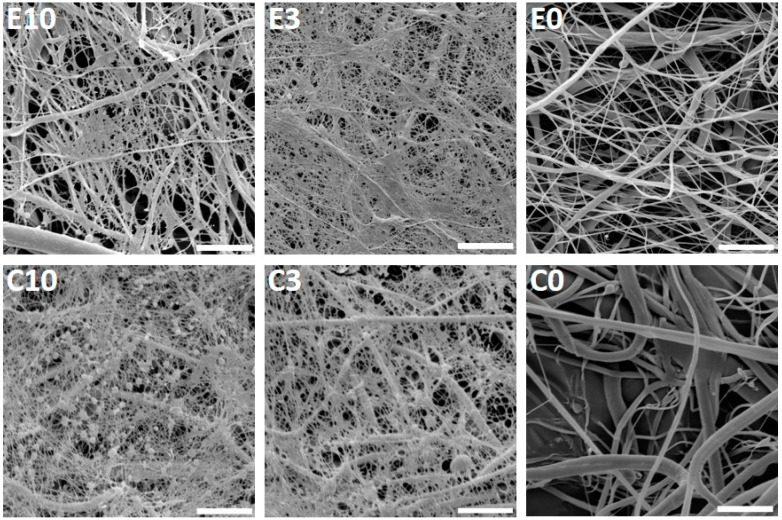
Scanning electron microscopy of the electrospun and centrifugally spun poly-ε-caprolactone (PCL) nanofibrous samples with adhered platelets. The images acquired on day 1 illustrate the fibrin network formed on the surface of the two samples with the highest platelet concentrations (**E10**, **C10**, **E3**, and **C3**) and the electrospun (**E0**) and centrifugally spun (**C0**) control samples. In the scaffold abbreviation, E stands for electrospun samples, C for centrifugally spun samples and the number indicates the fold change of the physiological concentration of platelets (300 × 10^9^ platelets/L). Magnification 6000×; scale bar 10 µm.

Furthermore, the fibrous scaffolds were functionalized with platelets and their morphology was examined using SEM (Figure 2). The acquired images showed a fine fibrin network formed on the electrospun and centrifugally spun scaffolds, with the two highest platelet concentrations (samples E10, C10, E3 and C3). On the samples with the lower platelet concentrations, the fibrin network was very scarce. The presence of the fibrin network was decreasing with the decreasing platelet concentration, and on the samples E0.1 and C0.1, no fibrin network was detected.

On the centrifugally spun samples, there were more platelets embedded within the fibrin network. The platelets showed morphology typical for their activated state-spread platelets with filopodia.

### 3.2. Characterization of the Platelet-Derived Bioactive Molecules

**Figure 3:**
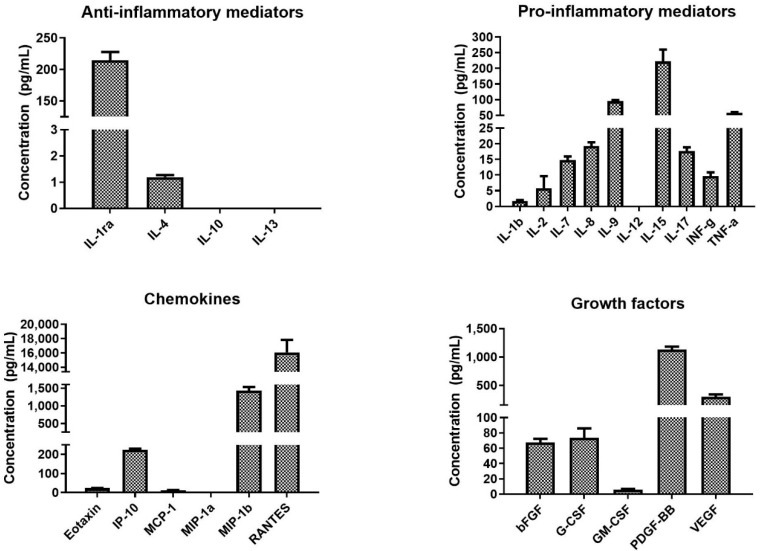
Concentration of the assayed bioactive molecules in the platelet concentrate. The bioactive molecules were determined using multiplexed assay.

The concentrations of bioactive molecules contained in the platelets were determined using X-MAP (Multi-Analyte Profiling) technology. The obtained results are shown in Figure 3 and the obtained values are summarized in Supplementary Table S1. The acquired data confirmed that platelets contained various pro- and anti-inflammatory cytokines, chemokines, and growth factors. The most abundantly present bioactive molecules were chemokine RANTES and PDGF-BB. Furthermore, the platelet lysate contained a high level of TSP-1 (1.9 ± 0.7 µg/mL) and TGF-β1 (59.3 ± 2.6 ng/mL). The data are included in Supplementary Table S1.

### 3.3. Release Characteristics of the Bioactive Molecules

**Figure 4:**
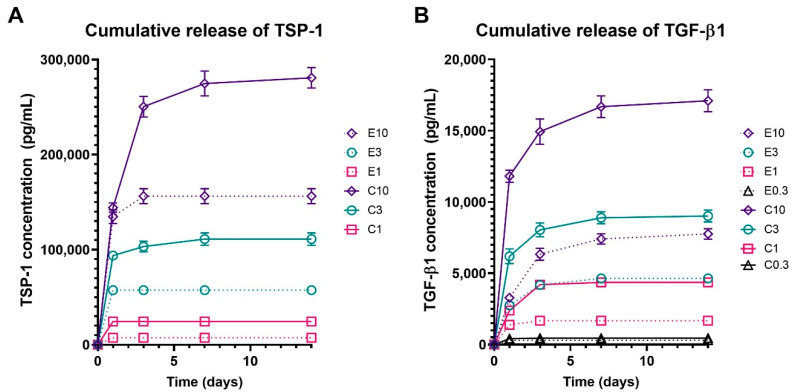
Cumulative release of thrombospondin-1 (TSP-1) and transforming growth factor beta (TGF-β1) from the platelet functionalized electrospun and centrifugally spun samples. In the scaffold abbreviations, E stands for electrospun samples, C for centrifugally spun samples and the number indicates the fold change of the physiological concentration of platelets (300 × 10^9^ platelets/L). Only data for the samples with analyte concentration above the detection limit of the assay are shown. (**A**)—Cumulative release of TSP-1 from the electrospun (E10, E3, E1) and centrifugally spun (C10, C3, C1) samples. (**B**)—Cumulative release of TGF-β1 from the electrospun (E10, E3, E1, and E0.3) and centrifugally spun (C10, C3, C1, and C0.3) samples.

To characterize the release of bioactive molecules from the functionalized samples, levels of TSP-1 and TGF-β1 released from the electrospun and centrifugally spun scaffolds were detected. TSP-1 and TGF-β1 were chosen as models of large and small molecules, respectively. Both of them are bioactive molecules abundant in platelets, fostering the proliferative processes during wound healing. The obtained data are shown in Figure 4. Detectable amounts of TSP-1 were only present in the samples with the three highest concentrations of platelets (E10 and C10, E3 and C3, and E1 and C1). The cumulative release of TSP-1 (Figure 4A) showed that the adhesion of platelets to the centrifugally spun scaffolds resulted in an approximately two-fold increase in the TSP-1 amount, when compared to the respective electrospun scaffolds. In the centrifugally spun sample with the highest concentration of platelets (C10), a significant amount of TSP-1 was detected, even on day 14. On the contrary, all of the TSP-1 was released from all the electrospun samples by day 3 of the experiment. Furthermore, the release of TGF-β1 from the platelet-functionalized scaffolds was investigated (Figure 4B). In contrast to the results observed in the TSP-1 release study, a significant amount of TGF-β1 was detected, even in the samples E0.3 and C0.3. The data followed a similar trend when compared to the TSP-1 data. There was a significantly higher concentration of detected TGF-β1 on the centrifugally spun scaffolds when compared to the respective electrospun scaffolds. Furthermore, in the higher tested platelet concentrations (C10, C3), the two-fold increase seen in TSP-1 was also observed in the case of TGF-β1.

Based on the acquired data, the half-time release of the investigated growth factors was determined. The half-time release of TSP-1 from the platelet-functionalized samples ranged from 0.5 day to almost 1 day and is shown in Table 2. The graphs showing the relative release of TSP-1 and TGF-β1 are shown in Supplementary Figure S1.

The half-time release detected in TGF-β1 was generally longer when compared to the detected levels of TSP-1.

### 3.4. Melanocytes Seeded on the Platelet-Functionalized Samples

**Figure 5:**
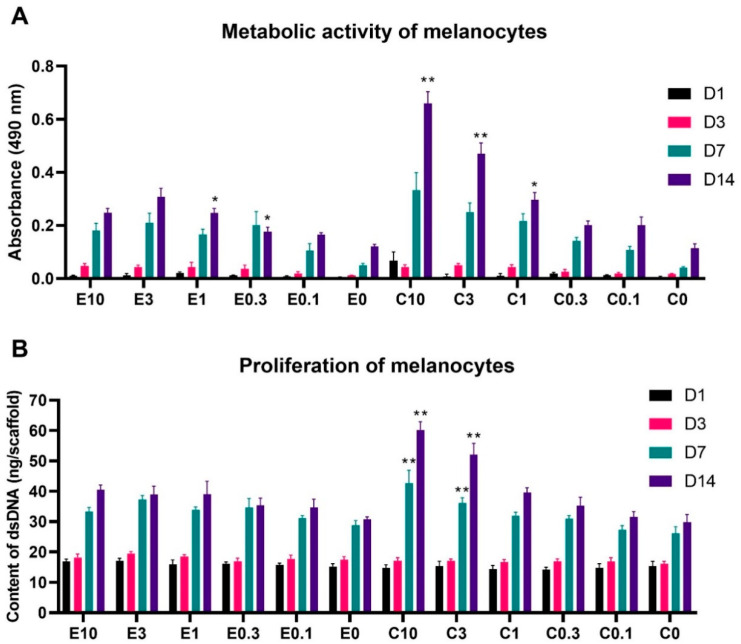
Metabolic activity and proliferation of melanocytes seeded on the electrospun and centrifugally spun scaffolds. (**A**)—Metabolic activity of melanocytes was determined by the MTS assay. (**B**)—Proliferation of melanocytes determined by dsDNA staining. For the sake of clarity, statistical significance is only included if the detected metabolic activity/proliferation on the respective scaffold was significantly higher when compared to all the samples functionalized with a lower concentration of platelets and the control sample containing no platelets. The data, including the statistical analysis in its entirety, are presented in Supplementary Figure S1. The level of significance was set at *p* < 0.001. In the scaffold abbreviations, E stands for electrospun samples, C for centrifugally spun samples and the number indicates the fold change of the physiological concentration of platelets (300 × 10^9^ platelets/L).

To test the biocompatibility of the scaffolds, the metabolic activity and proliferation of melanocytes seeded on platelet-functionalized scaffolds was determined. The metabolic activity of the melanocytes is shown in Figure 5A. For simplicity and clarity, the graphs only contain statistical significance if the detected metabolic activity on the sample was statistically significantly higher than all the samples with lower platelet concentrations. The graphs containing full statistical analysis are given in Supplementary Figure S2.

The acquired data showed a clear tendency of a dose-dependent effect of the platelet concentration, adsorbed to the scaffolds. On the electrospun scaffolds, the bioactive compounds released from the platelets significantly improved the melanocyte metabolic activity, in comparison to the plain PCL control (E1) from day 3 of the experiment. By the end of the experiment, the three highest platelet concentrations (samples E10, E3, and E1) significantly improved melanocyte metabolic activity, in comparison to the below-physiologic platelet concentration, with the sample E3 showing the highest metabolic activity. Furthermore, even the below-physiologic platelet concentrations (E0.3 and E0.1) resulted in improved melanocyte metabolic activity, when compared to the E0 control sample.

Similarly, as in the case of the electrospun scaffolds, the metabolic activity of melanocytes seeded on the centrifugally spun scaffolds, was platelet concentration dependent. In contrary to the below-physiological platelet concentrations (C0.1 and C0.3), the melanocyte metabolic activity was significantly improved in comparison to the C0 control from day 3. By the end of the experiment, the highest melanocyte metabolic activity was detected on the scaffold with the highest platelet concentration (C10), followed by the second highest platelet concentration (C3) and the physiological platelet concentration (C1).

The overall statistical analysis of all the obtained data (data not shown in the graph) showed there were no statistically significant differences between the electrospun and centrifugally spun samples on days 1, 3, and 7. However, on day 14, the metabolic activity of melanocytes seeded on the C10 scaffolds was significantly improved not only in comparison to all the other tested centrifugally spun scaffolds, but also in comparison to all the tested electrospun scaffolds. Furthermore, even the metabolic activity of melanocytes seeded on the second highest tested concentration of platelets adhered to the centrifugal scaffold (C3) was significantly improved in comparison to all the tested electrospun samples. These differences were levelled out at the physiological platelet concentration, as there was no significant difference in the metabolic activity of melanocytes seeded on the C1 and E1 samples. Furthermore, the morphology of the electrospun and centrifugally spun nanofibrous samples did not seem to affect the melanocyte metabolic activity, as there were no significant differences detected between the plain PCL control samples E0 and C0.

A similar trend was observed in the melanocyte proliferation data shown in Figure 5B (full statistical analysis shown in Supplementary Figure S3). There were only moderate statistically significant differences in the proliferation of melanocytes seeded on the electrospun scaffolds, with the E3 scaffold achieving the highest proliferation on day 7 of the experiment. Furthermore, the scaffolds with the three highest platelet concentrations (E10, E3, and E1) reached a significantly improved proliferation, in comparison to the E0 sample on day 14. The differences in melanocyte proliferation were more pronounced on the centrifugally spun samples. Until day 3, there were no statistically significant differences in melanocyte proliferation. However, on day 7, the proliferation of melanocytes seeded on the centrifugally spun scaffolds with the highest platelet concentration (C10), was significantly higher when compared to all the other centrifugally spun scaffolds, followed in the trend by the second highest platelet concentration (C3). The same trend was observed on day 14 of the experiment. The melanocyte proliferation on samples C1 and C0.3 was improved, compared to the plain PCL control C0. However, on the sample functionalized with the lowest platelet concentration (C0.1), no improvement in melanocyte proliferation was achieved. The overall statistical analysis (data not shown in the graph) confirmed the trend observed in the metabolic activity data. On day 7, the metabolic activity of melanocytes seeded on the centrifugally spun C10 scaffold showed the highest proliferation, when also compared to all the centrifugally spun and electrospun scaffolds. Furthermore, the response was further observed on day 14, when the C10 and the C3 samples showed a superior proliferation when compared to all the electrospun samples (in addition to the centrifugally spun samples). Similarly, there was no difference in the proliferation of melanocytes seeded on the E0 or C0 control samples, suggesting the sole effect of the platelet-derived molecules on the behavior of melanocytes, regardless of the scaffold morphology.

Melanin synthesized on the electrospun and centrifugally spun scaffolds was visible by the naked eye and detected using photography (Supplementary Figure S4). Since day 7, melanin deposits were visible on the electrospun and centrifugally spun scaffolds. The melanin synthesis was more pronounced and uniform on the centrifugally spun scaffolds, when compared to the electrospun scaffolds. There was very scarce melanin synthesis on the PCL controls (E0, C0) without any functionalization by platelets.

**Figure 6:**
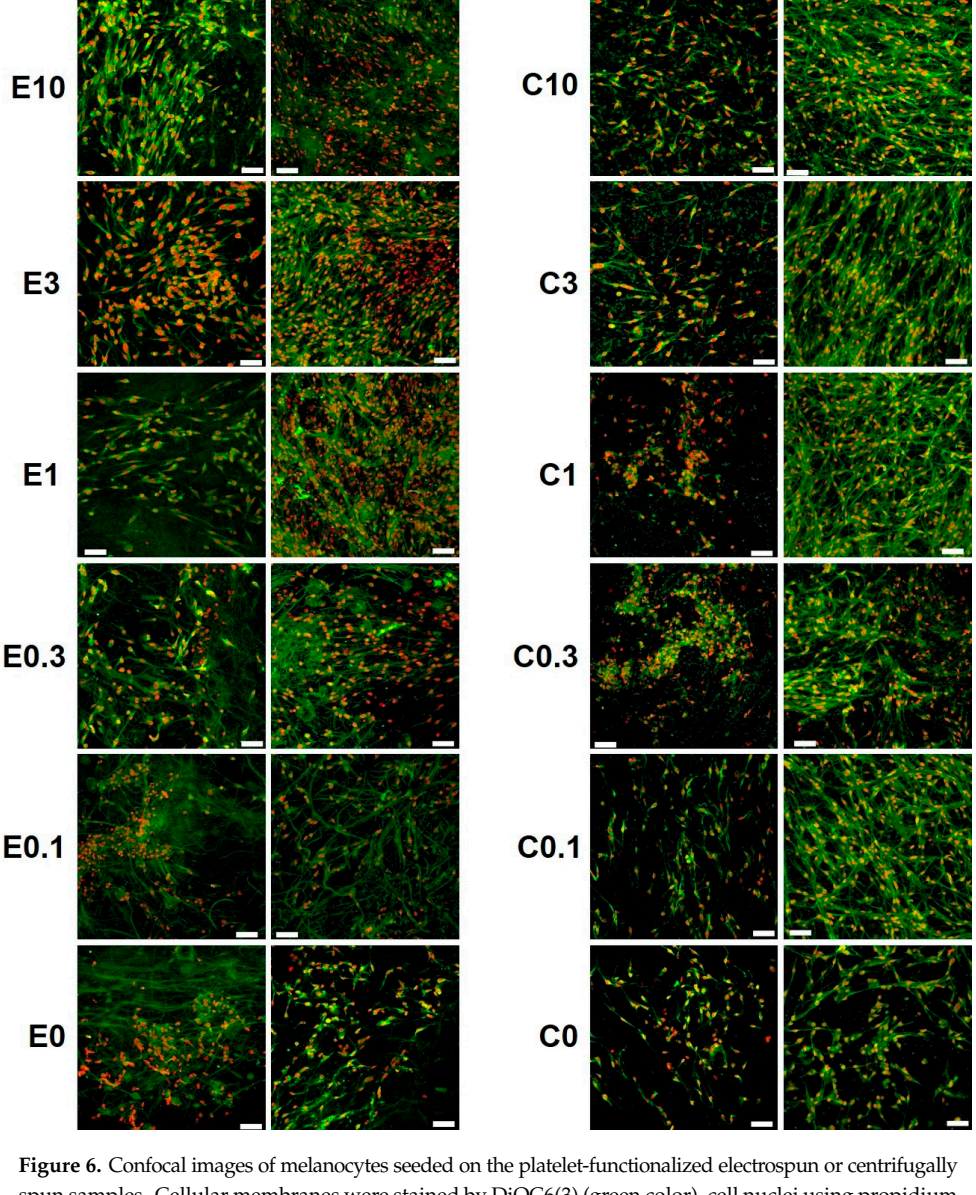
Confocal images of melanocytes seeded on the platelet-functionalized electrospun or centrifugally spun samples. Cellular membranes were stained by DiOC6(3) (green color), cell nuclei using propidium iodide (red color). For each sample, a representative image from day 7 and day 14 is given. In the scaffold abbreviations, E stands for electrospun samples, C for centrifugally spun samples and the number indicates the fold change of the physiological concentration of platelets (300 × 10^9^ platelets/L). Objective = 20×, scale bar 50 µm.

The morphology of melanocytes seeded on the platelet-functionalized samples was visualized using confocal microscopy (Figure 6). The confocal microscopy images confirmed the other obtained data. A significant increase in cell numbers was observed from day 7 to day 14, on the electrospun and the centrifugally spun nanofibrous scaffolds. There was an obvious difference in cell numbers on the platelet-functionalized samples and on the PCL control, on both the electrospun and the centrifugally spun scaffolds. Furthermore, the melanocytes cultured on the centrifugally spun scaffolds showed a more dendritic morphology and formed a more complex network, in comparison to the melanocytes cultured on the more 2D-like electrospun morphology.

## 4. Discussion

Vitiligo is the most commonly acquired depigmentation skin disorder. The currently available treatment modalities are not satisfactory, and they often suffer from poor patient compliance due to their long-term nature. Therefore, PRP has recently attracted much attention, as its beneficial effects have already been used in dermatology for skin rejuvenation and acne scar treatments [33]. The bioactive substances contained in PRP not only inhibit the inflammation processes but stimulate the regenerative processes that are also necessary to restore the normal melanocyte function in the epidermis [34].

PRP in vitiligo treatment was first mentioned by Lim et al., who concluded that intradermal PRP injections were ineffective in 20 studied patients [35]. Several clinical trials have subsequently been performed, to evaluate the efficacy of PRP application in combination with conventional therapy. Ibrahim et al. compared intradermal PRP in combination with NB-UVB phototherapy. Their study demonstrated that the combination resulted in significantly improved re-pigmentation over a short period of time, when compared to the sole phototherapy group (75% of patients in the PRP+NB-UVB group achieved an excellent or good response, in comparison to no response in the NB-UVB group) [9]. Similarly, the intradermal PRP application in combination with fractional CO_2_ laser therapy [10,36] and excimer laser use [37], proved to be superior when compared to the respective laser monotherapy. The combination of laser/NB-UVB phototherapy and PRP application reduces phototherapy exposure [37], improves re-pigmentation and thus patient compliance. Saify et al. studied the route of administration of PRP. They compared the topical administration of PRP following micro-needling, intradermal injections and intravenous administration. According to their results, topical administration resulted in a good color match and promoted repigmentation [38]. Therefore, a drug delivery system that could prolong the topical delivery of growth factors following a micro-needling treatment would be beneficial. The aim of this study was to develop a simple and effective drug delivery system for platelet-derived bioactive molecules.

Firstly, the morphology of the prepared fibrous scaffolds was characterized, as differing morphology, e.g., fiber diameter and roughness, may affect cell behavior and platelet activation. The detected mean diameter of the centrifugally spun fibers was higher in comparison to the diameters obtained via electrospinning, with a greater variance that is due to the less controlled fabrication procedure; this is in accordance with previous studies [39,40]. The fiber diameter above 2 µm and mainly above 5 µm significantly increased platelet adhesion, activation and thrombin formation on electrospun vascular grafts [41]. Generally, both types of scaffolds mimic the native extracellular matrix (ECM) owing to their porosity, pore interconnection and fibrous structure. However, the electrospun fibrous layer is more planar, forming a rather 2D-like structure, in comparison to the centrifugally spun fluffy 3D-like scaffolds with higher porosity, improving cell penetration that is impeded to some degree in the 2D-like electrospun layers [25,39]. The µCT evaluation showed a higher number of larger pores in the centrifugally spun samples, when compared to the electrospun samples. Similar results were described by Krifa et al., who compared fiber diameter and pore size in electrospun and centrifugally spun membranes made of nylon 6 [42]. According to their results, centrifugally spun fibers were looser, had higher fiber diameter and broader diameter distribution. A study performed on PCL confirmed that a centrifugally spun fibrous scaffold showed higher porosity, pore size and fiber diameter [25]. It has been shown that the more open morphology of the centrifugally spun samples is a desirable feature in tissue engineering applications, as it fosters cell penetration into the deeper layers of the scaffold and its colonization [43]. The higher mean pore size relevant to the size of platelets in combination with the results from growth factor release studies, indicate higher platelet adhesion on the centrifugally spun nanofibers. The open structure of the centrifugally spun scaffolds with higher pore size enabled deeper penetration of the platelets and resulted in an increased adsorption surface. Therefore, the platelets adsorbed on the centrifugally spun fibers released a larger amount of growth factors.

The higher metabolic activity of platelets on the surface of the centrifugally spun samples suggested a more pronounced adhesion of platelets to the samples. The performed SEM analysis demonstrated the presence of a fine fibrin network on the surface of the fibrous samples, functionalized with the highest concentrations of platelets. A similar fibrin network was observed in other studies, evaluating the positive effect of combining platelets and nanofibrous scaffolds [25,44,45]. It has been shown that the structure of the fibrin network, in terms of diameter, branching and density, may be influenced by various factors including pH and calcium, thrombin or fibrinogen concentration [46]. In our previous study, we even observed a fibrin network had formed on the PCL scaffolds functionalized with a lower platelet concentration than in this study [45]. The explanation for this may be due to the decreased concentration of fibrinogen in the platelet concentrate solutions, as fibrinogen is mainly present in plasma. Fibrinogen is the main factor affecting the fibrin matrix composition; its concentration is proportional to the fibrin stiffness and fibrin network degradation rate. It was determined that the platelet concentration affects the formation of the fibrin network; higher platelets concentrations resulted in a finer (lower fiber diameter) fibrin network [47]. Several studies were conducted to investigate the effect of fibrin stiffness on cell proliferation; the results showed for example that keratinocytes preferred softer matrices, whereas fibroblasts preferred stiffer fibrin networks [48]. The mechanical properties and rapid breakdown of fibrin-only matrices described by several studies [49,50] limit the use of fibrin in some of the tissue engineering applications. Combining the fibrin network with nanofibers improves the handling of the scaffolds and prolongs their stability. The SEM analysis of a similar drug delivery system showed that platelets and fibrin network residual were present on the nanofibrous samples, even on day 14 [25].

Not only does the fibrin network provide a scaffolding system for cells, it also serves as a reservoir of growth factors released from the activated platelets and affects their spatiotemporal release [48,51]. Several growth factors have been shown to interact with the components of the fibrin matrix, such as VEGF [52], bFGF [53,54] and TGF-β1 [55]. In our study, the half-time of TGF-β1 release was generally longer when compared to the detected levels of TSP-1. These findings indicated a stronger interaction of TGF-β1, with the fibrin matrix formed on the surface of the nanofibers. This is in accordance with the findings of a study that investigated the binding of several growth factors (TGF-β1 included) to fibrin. In the study, the growth factors were combined with a fibrinogen solution; the fibrinogen was polymerized using thrombin and factor XIII. The concentration of growth factors released from the fibrin matrix was determined. The study showed a strong interaction of TGF-β1 with fibrinogen, via its heparin-binding domain. In the study, 50% of the contained TGF-β1 was released after 1.5 days, which is in accordance with our results [56]. Thus, by exploitation of a naturally occurring phenomenon, the release of bioactive molecules may be controlled, and a simple drug delivery system may be developed.

To test the biocompatibility of the scaffolds and the response of melanocytes to the bioactive molecules released from the drug delivery system, the scaffolds were seeded with melanocytes and evaluated. The melanocyte metabolic activity and proliferation was significantly affected by the presence of platelets and the platelet-derived bioactive molecules in a concentration-dependent manner; both measures increased with the increasing concentration of platelets. The best results were obtained in the centrifugally spun samples with the highest concentration of platelets (the highest tested concentration of platelet-derived molecules, C10). Nanofibers made of PCL, using electrospinning, were successfully used for the melanocyte culture [45,57,58]. Savkovic et al. showed PCL nanofibers to be superior to a 2D cell culture substrate, in terms of melanin synthesis [57]. The functionalization of PCL nanofibers with platelets led to improvements of cell metabolic activity and proliferation, not only in melanocytes but also in keratinocytes and fibroblasts [45]. Such pleotropic effects of PRP may be very useful in vitiligo therapy, as keratinocytes and fibroblasts were also implicated in its etiopathogenesis [9]. The acquired data further showed that the metabolic activity and proliferation of melanocytes was not affected by the morphology of the scaffolds, as there were no statistically significant differences between the electrospun and centrifugally spun control samples. However, the confocal microscopy study revealed changes in the morphology of melanocytes, cultured on the centrifugally spun samples. Similar changes in melanocyte morphology in a platelet lysate-enriched culture medium were observed [58]. These changes may be attributed to the high PDGF-BB contained in the platelets, as the PDGF-BB has been shown to stimulate melanogenesis, dendritogenesis, and the differentiation of human melanocytes [59].

The principles of tissue engineering also became apparent in vitiligo treatment. To improve the melanocyte transplantation outcomes, a “cellular patch” concept was proposed. Such a patch consisting of a suitable biomaterial, would allow in vitro expansion of the harvested melanocytes [60]. Accordingly, human amniotic membrane [61] or various polymer films consisting of poly (DL-lactic acid) [62], plasma-treated polyvinyl chloride and silicone [63] or polystyrene [64] were investigated in vitro and in clinical practice. Therefore, the active stimulation of melanocyte proliferation and mechanical support provided by our system would be beneficial in clinical applications. Furthermore, given the cytokines, growth factors, and other bioactive molecules, the fibrous scaffolds functionalized with platelets could serve as a cell-free dressing with sustained delivery of biomolecules to the site of interest. This requires further investigation.

## 5. Conclusions

The PRP has proven to be useful in combination vitiligo therapy, as it represents a very safe and cheap regenerative therapy. Different drug delivery systems may improve the current re-pigmentation outcomes. The results of the study showed that the centrifugally spun scaffolds yielded almost a two-fold increase in the number of platelets adhered to the surface of the fibrous mesh, prolonging the delivery of platelet-derived bioactive molecules. A strong concentration-dependent behavior of melanocytes seeded on the platelet-functionalized scaffolds was observed, as with the increasing concentration of the bioactive molecules, the melanocyte response was also increasing. The most pronounced response of melanocytes was observed in the highest tested concentration of bioactive molecules.

Overall, the unique properties of nanofibers allow them to form non-woven textiles, with sufficient mechanical capacity, to be utilized as wound dressings in clinical practice. The key innovation for vitiligo treatment of the proposed scaffold system is its active stimulation of melanocytes by platelets as a source of growth factors. The system is overcoming other application systems for platelet delivery by their immobilization. Upon simple spraying on the skin, the platelets would be rapidly washed from the application site. If combined with coatings (i.e., films), the structure of the film would not provide sufficient porosity to enable optimal transition of moisture and fluids, resulting in a suboptimal skin reaction. Moreover, our group intensively builds on the industrialization of nanofiber production, leading to commercial viability of the proposed solution for topical administration, due to its simplicity and on-site preparation provision.

## Figures and Tables

**Table 1:** The composition of the electrospun and centrifugally spun samples functionalized with platelets. In the scaffold abbreviations, E stands for electrospun samples, C for centrifugally spun samples and the number indicates the fold change of the physiological concentration of platelets (300 × 10^9^ platelets/L).

	Sample	Platelet Concentration
**Electrospun scaffolds**		
10× physiological concentration	E10	3000 × 10^9^ platelets/L
3× physiological concentration	E3	900 × 10^9^ platelets/L
Physiological concentration	E1	300 × 10^9^ platelets/L
3× diluted physiological concentration	E0.3	100 × 10^9^ platelets/L
10× diluted physiological concentration	E0.3	30 × 10^9^ platelets/L
Control	E0	No platelets
**Centrifugally spun scaffolds**		
10× physiological concentration	C10	3000 × 10^9^ platelets/L
3× physiological concentration	C3	900 × 10^9^ platelets/L
Physiological concentration	C1	300 × 10^9^ platelets/L
3× diluted physiological concentration	C0.3	100 × 10^9^ platelets/L
10× diluted physiological concentration	C0.1	30 × 10^9^ platelets/L
Control	C0	No platelets

**Table 2:** Half-time of TSP-1 and TGF-β1 release from the platelet-functionalized samples. The table represents the time to the release of 50% of the contained TSP-1 and TGF-β1 in days. In the scaffold abbreviations, E stands for electrospun samples, C for centrifugally spun samples and the number indicates the fold change of the physiological concentration of platelets (300 × 10^9^ platelets/L).

Sample	Half-Time of TSP-1 Release (Days)	Half-Time of TGF-β1 Release (Days)
C10	0.98	0.72
C3	0.59	0.73
C1	0.5	0.91
E10	0.57	1.39
E3	0.50	0.84
E1	0.50	0.61

## Supplementary Materials

The following are available online at https://www.mdpi.com/2079-4991/10/9/1801/s1, Table S1: Concentration of the determined analytes in the platelet concentrate. LOQ stands for limit of quantification. Figure S1: Relative release of TSP-1 (A) and TGF-β1 (B) from the electrospun (E) and centrifugally spun (C) scaffolds. Figure S2: Metabolic activity of melanocytes seeded on the electrospun and centrifugally spun scaffolds. A—Metabolic activity of melanocytes seeded on the electrospun PCL scaffolds (statistical analysis *p* < 0.05). B—Metabolic activity of melanocytes seeded on the centrifugally spun PCL scaffolds (statistical analysis *p* < 0.05). The level of significance is denoted by the numbers above the bars in the graph. E stands for electrospun, C stands for centrifugally spun and the number indicates the fold change of the physiological concentration of platelets (300 × 109 platelets/L). Figure S3: Proliferation of melanocytes seeded on the electrospun and centrifugally spun scaffolds. A—Proliferation of melanocytes seeded on the electrospun and centrifugally spun scaffolds. A—Proliferation of melanocytes seeded on the electrospun PCL scaffolds (statistical analysis *p* < 0.05). B—Proliferation of melanocytes seeded on the centrifugally spun PCL scaffolds (statistical analysis *p* < 0.05). The level of significance is denoted by the numbers above the bars in the graph. E stands for electrospun, C stands for centrifugally spun and the number indicates the fold change of the physiological concentration of platelets (300 × 109 platelets/L). Figure S4: Visualization of melanin synthesis on the electrospun and centrifugally spun fibrous scaffolds.
