# Supplementary material for: A Simple Drug Delivery System for Platelet-Derived Bioactive Molecules, to Improve Melanocyte Stimulation in Vitiligo Treatment
Source: Nanomaterials (Basel). 2020 Sep 10;10(9):1801. doi: 10.3390/nano10091801 (PMC7559479; doi:10.3390/nano10091801)
Supplement: Supplementary file 1 [file nanomaterials-10-01801-s001.pdf]

Article

A Simple Drug Delivery System for Platelet-Derived Bioactive Molecules, to Improve Melanocyte Stimulation in Vitiligo Treatment

Table S1. Concentration of the determined analytes in the platelet concentrate. LOQ stands for limit of quantification.

Pro-inflammatory cytokines		
	IL-1b	1.7 ±0.3 pg/mL
	IL-2	7.1 ±1.2 pg/mL
	IL-6	15.6 ±4.0 pg/mL
	IL-7	14.8 ±1.1 pg/mL
	IL-8	19.2 ±1.2 pg/mL
	IL-9	95.4 ±3.1 pg/mL
	IL-12	<LOQ
	IL-15	223.3 ±15.1 pg/mL
	IL-17	17.7 ±1.1 pg/mL
	IFN-γ	9.7 ±1.2 pg/mL
	TNF-α	57.2 ±3.5 pg/mL
Anti-inflammatory cytokines		
	IL-1ra	214.8± 13.1 pg/mL
	IL-4	1.2 ±0.1 pg/mL
	IL-5	39.4 ±5.3 pg/mL
	IL-10	<LOQ
	IL-13	<LOQ
Chemokines		
	Eotaxin	23.3 ±0.8 pg/mL
	IP-10	225.0 ±5.4 pg/mL
	MCP-1	12.3 ±1.2 pg/mL
	MIP-1a	1.3 ±0.1 pg/mL
	MIP-1b	1,426.8 ±105.2 pg/mL
	RANTES	15,982.6 ±1,729.8 pg/mL
Growth factors		
	IGF-1	32.4 ng/mL
	TGF-β1	17,569.9 ±1,507.8 pg/mL
	bFGF	67.7 ±4.7 pg/mL
	G-CSF	73.6 ±12.5 pg/mL
	GM-CSF	6.0 ±1.0 pg/mL
	PDGF-BB	1,125.5 ±55.6 pg/mL
	VEGF	294.7 ±48.1 pg/mL

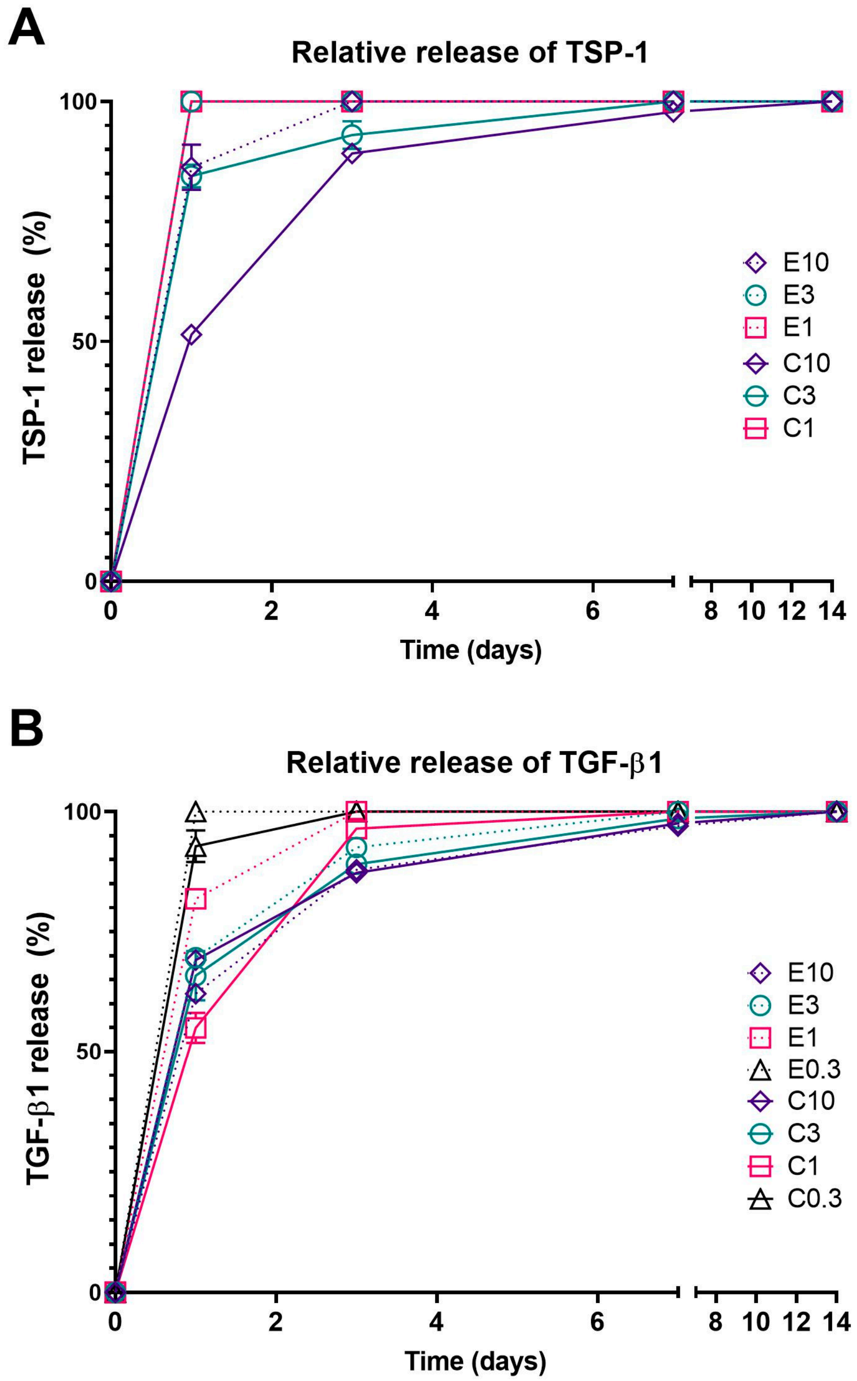

Figure S1 Relative release of TSP-1 (A) and TGF-β1 (B) from the electrospun (E) and centrifugally spun (C) scaffolds.

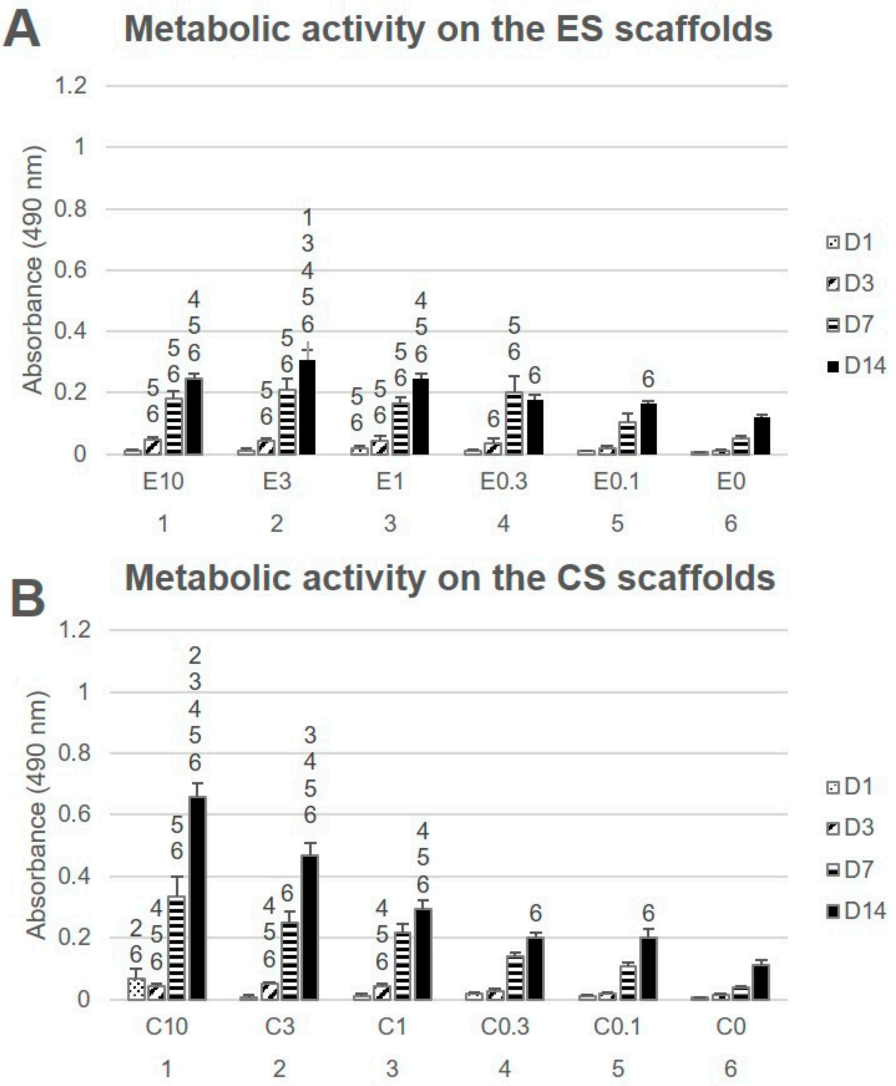

Figure S2 Metabolic activity of melanocytes seeded on the electrospun and centrifugally spun scaffolds. A – Metabolic activity of melanocytes seeded on the electrospun PCL scaffolds (statistical analysis p<0.05). B – Metabolic activity of melanocytes seeded on the centrifugally spun PCL scaffolds (statistical analysis p<0.05). The level of significance is denoted by the numbers above the bars in the graph. E stands for electrospun, C stands for centrifugally spun and the number indicates the fold change of the physiological concentration of platelets (300x109 platelets/L).

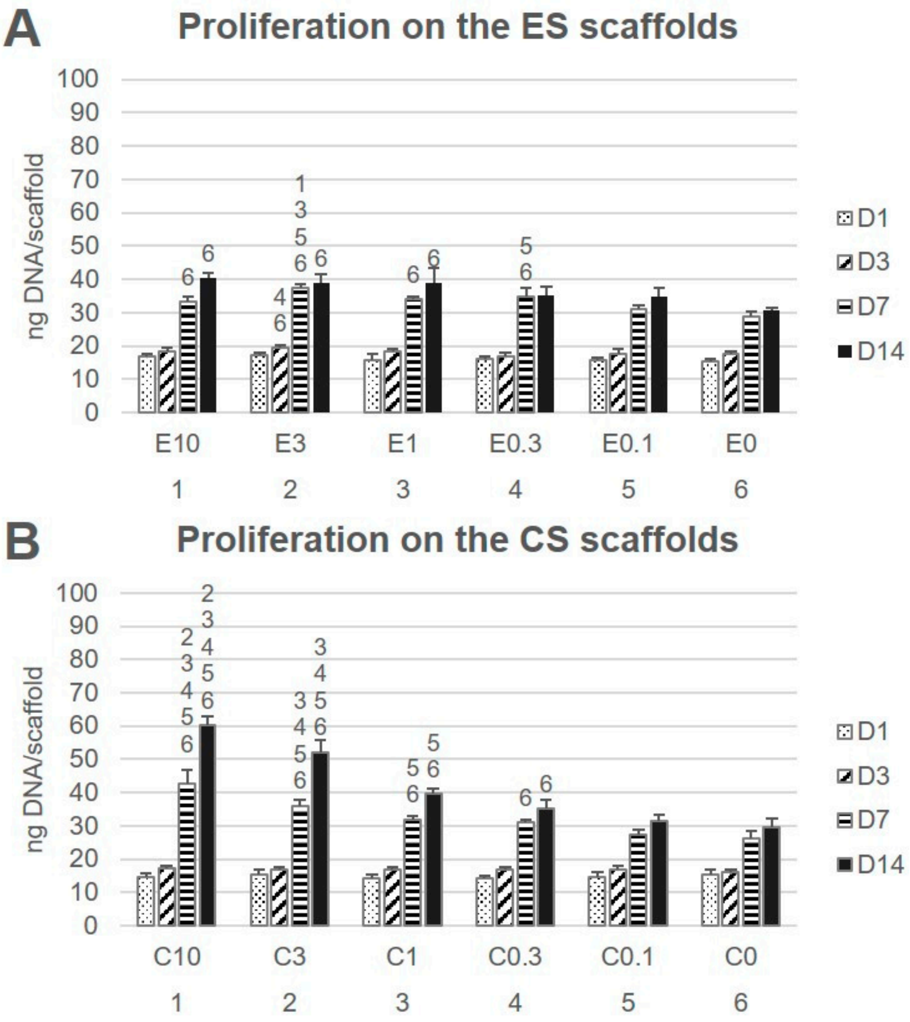

Figure S3 Proliferation of melanocytes seeded on the electrospun and centrifugally spun scaffolds. A – Proliferation of melanocytes seeded on the electrospun PCL scaffolds (statistical analysis p<0.05). B – Proliferation of melanocytes seeded on the centrifugally spun PCL scaffolds (statistical analysis p<0.05). The level of significance is denoted by the numbers above the bars in the graph. E stands for electrospun, C stands for centrifugally spun and the number indicates the fold change of the physiological concentration of platelets (300x109 platelets/L).

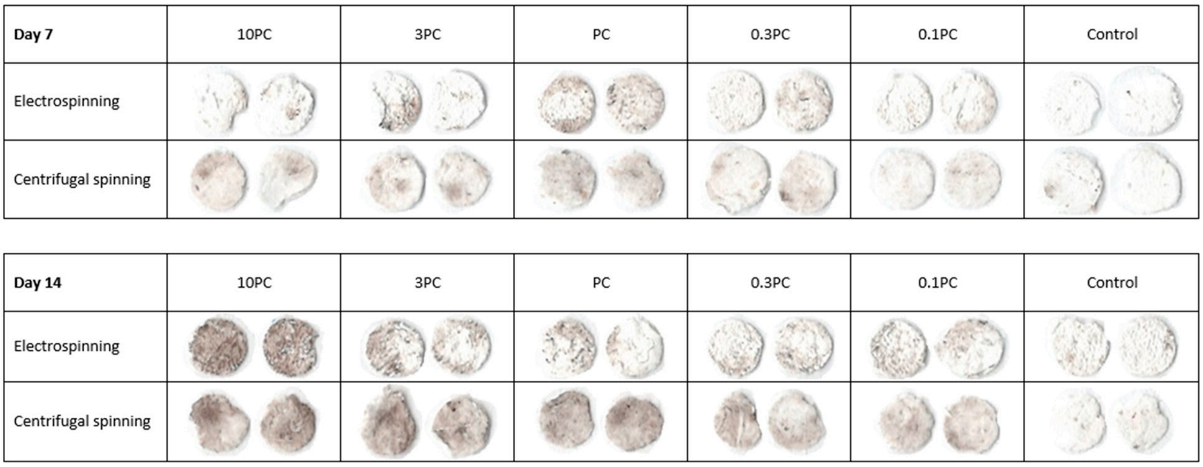

Figure S4 Visualization of melanin synthesis on the electrospun and centrifugally spun fibrous scaffolds.
